# Evaluation of the Contribution of the *EYA4* and *GRHL2* Genes in Korean Patients with Autosomal Dominant Non-Syndromic Hearing Loss

**DOI:** 10.1371/journal.pone.0119443

**Published:** 2015-03-17

**Authors:** Ye-Ri Kim, Min-A Kim, Borum Sagong, Seung-Hyun Bae, Hyo-Jeong Lee, Hyung-Jong Kim, Jae Young Choi, Kyu-Yup Lee, Un-Kyung Kim

**Affiliations:** 1 Department of Biology, College of Natural Sciences, Kyungpook National University, Daegu, Republic of Korea; 2 School of Life Sciences, BK21 Plus KNU Creative BioResearch Group, Kyungpook National University, Daegu, Republic of Korea; 3 Department of Otorhinolaryngology-Head and Neck Surgery, Hallym University College of Medicine, Anyang, Republic of Korea; 4 Department of Otorhinolaryngology, Yonsei University College of Medicine, Seoul, Republic of Korea; 5 Department of Otorhinolaryngology-Head and Neck Surgery, School of Medicine, Kyungpook National University, Daegu, Republic of Korea; National Eye Institute, UNITED STATES

## Abstract

*EYA4* and *GRHL2* encode transcription factors that play an important role in regulating many developmental stages. Since *EYA4* and *GRHL2* were identified as the transcription factors for the DFNA10 and DFNA28, 8 *EYA4* mutations and 2 *GRHL2* mutations have been reported worldwide. However, these genes have been reported in few studies of the Korean population. In this study, we performed a genetic analysis of *EYA4* and *GRHL*2 in 87 unrelated Korean patients with autosomal dominant non-syndromic hearing loss (NSHL). A total of 4 genetic variants in the *EYA4* gene were identified, including the 2 nonsense mutations p.S288X and p.Q393X. The novel mutation p.Q393X (c.1177C>T) resulted in a change in the codon at amino acid position 393 from a glutamine to a stop codon. The p.Q393X allele was predicted to encode a truncated protein lacking the entire C-terminal Eya homolog region (Eya HR), which is essential for the interaction with the transcription factor SIX3. The p.S288X (c.863C>A) mutation was found in a Korean family from a previous study. We analyzed p.S288X-linked microsatellite markers and determined that p.S288X might be a founder mutation and a hotspot mutation in Koreans. In *GRHL2*, a total of 4 genetic variants were identified, but none were associated with hearing loss in Korean patients. This suggests that *GRHL2* may not be a main causal gene for autosomal dominant NSHL in Korean patients. In conclusion, our data provide fundamental information to predict the genotypes of Korean patients diagnosed with autosomal dominant NSHL.

## Introduction

Transcription factors regulate the time and location of expression of thousands of genes; they are involved in DNA binding, cellular localization, protein stability and so on. Recently, transcription factors have been reported that regulate the proper development and functioning of the inner ear [1–3]. The *EYA4* (OMIM 601316) and *GRHL2* (OMIM 608641) genes are transcription factors related constitution of the organ of Corti [4, 5].

Vertebrate genomes encode four Eyes absent (EYA) proteins (EYA1–4), which consist of two domains, the C-terminal Eya homolog region (HR) and the N-terminal *transactivation* Eya variable region (VR) [6]. EYA proteins regulate the post-developmental function of the organ of Corti, DNA damage repair, cell survival in response to DNA damage, angiogenesis, and cancer metastasis [7, 8]. EYA4 maps to the autosomal dominant non-syndromic hearing loss (NSHL) locus DFNA10 on chromosome 6q23. The sole phenotype resulting from mutations in the EYA4 gene is hearing loss [4, 9]. To date, 8 mutations in the *EYA4* gene have been identified, including missense mutation, splicing mutation, small deletions, small insertions, and partial or complete truncation or deletion of the Eya HR domain (Table 1) [4, 10–14]. The onset of *EYA4* mutation-mediated hearing loss is remarkably broad, ranging from early childhood to adulthood, and hearing deficits are not stable until adulthood [15]. The hearing loss severity of all of patients with mutations was progressive, with a general trend of spontaneous evolution to a moderate or severe hearing impairment.

**Table 1:** Summary of *EYA4* gene mutations identified in previous and current studies.

Family origin	Location	Nucleotide change	Amino acid change	Severity	Domain	References
Korean	Exon 11	c.863C>A	p.S288X	Moderate	Eya VR	Baek *et al*., 2012
Korean	Exon 12	c.978C>G	p.F326L	Moderately severe	Eya VR	Choi *et al*., 2013
American	Exon 12	c.1026_1027dupAA	p.T343KfsX27	N/A*	Eya VR	Wayne *et al*., 2001
American	Exon 12	c.1048_1049dupAA	p.R352PfsX18	Mild to severe	Eya HR	Makishima *et al*., 2007
Hungarian	Exon 13	c.1115_1118dupTTGT	p.W374SfsX6	N/A*	Eya HR	Pfister *et al*., 2002
Korean	Exon 13	c.1177C>T	p.Q393X	Moderately severe	Eya HR	This study
Australian	Intron 14	c.1282–12T>A	p.E428FfsX5	Mild to severe	Eya HR	Hildebrand *et al*., 2007
Chinese	Exon 15	c.1301T>A	p.I411K	Mild to severe	Eya HR	Tan *et al*., 2014
Belgian	Exon 20	c.1759C>T	p.R587X	N/A*	Eya HR	Wayne *et al*., 2001

* N/A, Not available.

The *GRHL2* gene is responsible for autosomal dominant NSHL, affecting the DFNA28 locus. It encodes the Grainy head like 2 (GRHL2) protein, also known as transcription factor cellular promoter 2-like 3 (TFCP2L3), which is highly expressed in epithelial cells lining the cochlear duct, and not only plays a critical role in embryonic development but also maintains epithelial cells throughout life [16]. The gene is involved in otic development and hearing in animals, and *GRHL2* mutations are correlated with hearing loss in humans [16]. The DFNA28 locus was first associated with GRHL2 in mapping studies investigating 5 generations of a North American family affected with NSHL [16]. In the study, affected members had a c.1609_1610insC mutation in exon 13.

There is little research on *EYA4* and *GRHL2* in the Korean population. Therefore, we analyzed *EYA4* and *GRHL2* in patients with autosomal dominant NSHL to identify the effects of these transcription factors in Korean patients.

## Materials and Methods

### Subjects and clinical evaluation

A total of 87 unrelated subjects with autosomal dominant NSHL were recruited from the Department of Otorhinolaryngology–Head and Neck Surgery at Kyungpook National University Hospital in Daegu, Yonsei University Health System Hospital in Seoul, and Hallym University Hospital in Chuncheon, Korea. The sample included 33 male and 54 female patients, with an average age of 40.439 ranging from 3 months to 73 years. The hearing levels of all patients and normal controls were examined using pure-tone audiometry (PTA) [17]. PTA was calculated as the average threshold measured at 500, 1000, 2000, and 4000 Hz, and air-conduction threshold measurements were performed at 125–8000 Hz [18, 19].

One hundred unrelated Koreans with normal hearing were recruited from Kyungpook National University Hospital as normal controls. All of the participants provided written informed consent before the study, according to the protocol approved by the Ethics Committee of Kyungpook National University Hospital. If necessary, we requested samples of family members after informed consent was obtained.

### Molecular genetic analysis

The genomic DNA of 87 subjects and 100 normal controls was extracted from blood or buccal cells using a FlexiGene DNA Extraction Kit (Qiagen, Hilden, Germany). All of the subjects were examined for *EYA4* and *GRHL2* gene expression by Sanger sequencing. All 21 exons of *EYA4*, 16 exons of *GRHL2*, and intron–exon boundaries were amplified by polymerase chain reaction (PCR) using h-Taq DNA Polymerase (Solgent, Daejeon, Korea). These primers and PCR cycling information are available upon request. The amplified products were purified using shrimp alkaline phosphatase (USB, Cleveland, OH, USA) and exonuclease (USB, Cleveland, OH, USA), and were then sequenced using an ABI BigDye Terminator v3.1 Cycle Sequencing Kit (Applied Biosystems, Foster City, CA, USA). An ABI 3130XL genetic analyzer (Applied Biosystems, Foster City, CA, USA) was used to assay the products, and data were analyzed using ABI Sequencing Analysis v5.2 (Applied Biosystems, Foster City, CA, USA) and Chromas Pro v1.7.3 (Technelysium, Tewantin, QL, Australia). The sequencing data were then compared with the reference sequence using the Basic Local Alignment Search Tool (BLAST) on the National Center for Biotechnology Information (NCBI) website (http://www.ncbi.nlm.nih.gov). The dbSNP (http://www.ncbi.nlm.nih.gov/snp/) and the 1000 Genome Database (http://www.1000genomes.org/) were used to investigate the newly identified variants and the probable pathogenicity of detected variants.

### Short tandem repeat (STR) marker genotyping

Primers for 5 STR markers spanning the *EYA4* gene were designed by Map Viewer on the NCBI website (http://www.ncbi.nlm.nih.gov/mapview/). Primers designed from the genomic sequences available from the University of California-Santa Cruz (UCSC) Genome Browser (GRCh37/hg19). The sequences of the markers were D6S1603 (forward, 5´-ATT TTA TGC TTA AAG ATG CAG AA-3´; reverse, 5´-GTA CCT ACG GGT GCC G-3´), D6S262 (forward, 5´- ATT CTT ACT GCT GGA AAA CCA T-3; reverse, 5´- GGA GCA TAG TTA CCC TTA AAA TC-3´), D6S1038 (forward, 5´- TCC TGA ATT GTA CAC TTA AAA TGG-3´; reverse, 5´- GCA GGC ATA TTG CTG TTC TT-3´), D6S292 (forward, 5´- AAT TCA CAA GAC ACA ATC TCA G-3; reverse, 5´- AGA ACT AAA GTT GCC TGT TCN TGT A-3´), and D6S1564 (forward, 5´-ATT TTA TGC TTA AAG ATG CAG AA-3´; reverse, 5´-GGG GAC ACA GCC AAA-3´). These were amplified with e-Taq DNA Polymerase (Solgent, Daejeon, Korea). The PCR cycling and protocol information are available upon request. An ABI 3130XL genetic analyzer (Applied Biosystems, Foster City, CA, USA) was used to assay the products, and data were analyzed using GeneMapper v4.1 & Data Collection v3.1 (Applied Biosystems, Foster City, CA, USA).

### *In silico* predictions

We used three approaches including pathogenicity prediction programs, PolyPhen-2 (http://genetics.bwh.harvard.edu/pph2/), SIFT (http://sift.jcvi.org) and MutationTaster (http://mutationtaster.org) to predict whether a single nucleotide change in the exon 9 of the *GRHL2* gene may cause abnormal protein function. The result of PolyPhen-2 was a prediction of probably damaging or benign, with a numerical score ranging from 0.0 (benign) to 1.0 (damaging) [20]. The SIFT showed scores ranging from 0.0 (damaging) to 1.0 (benign) that were calculated using position-specific scoring matrices with Dirichlet priors [21]. The MutationTaster gave prediction as either ‘disease-causing’ or ‘polymorphism’ along with a P value [22]. We used two approaches to predict whether a single nucleotide change in the sequence of *EYA4* exon 7 or *GRHL2* exon 15 might cause abnormal splicing. Bioinformatic prediction tools were used to evaluate the effect of mutations. Predicted exonic splicing enhancer (ESE) motifs were screened using the ESEfinder web-interface (http://rulai.cshl.edu/tools/ESE), which provided a score based on the frequency of nucleotides at each position in the sequence of the motif specifically recognized by the four SR proteins SF2/ASF, SC35, SRp40, and SRp55. In addition, we used RESCUE-ESE (http://genes.mit.edu/burgelab/rescue-ese) to predict expected splicing enhancer alterations. This program calculates the total number of exonic splicing silencers (ESSs), ESEs, and their ratio.

## Results

### Genetic analysis of *EYA4*

**Fig 1:**
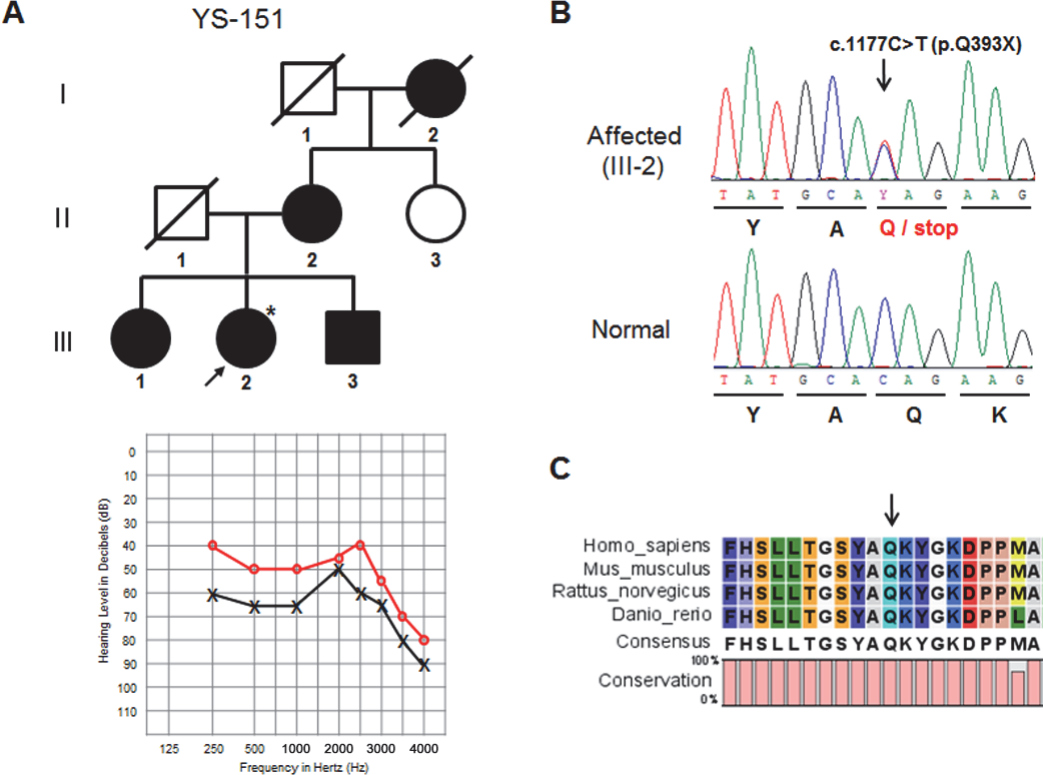
Mutation analysis of the *EYA4* gene in the YS-151 family. (A) Pedigree of a Korean family with autosomal dominant inheritance (upper panel). A three-generation pedigree that includes 8 members is presented. The filled symbols and open symbols indicate affected and unaffected individuals, respectively. The arrow designates the proband. Pure tone audiogram for the left and right ears of the YS-151 patient (III-2) (lower panel). The circles and crosses indicate unmasked air conduction thresholds for the right and left ears, respectively. (B) DNA sequencing analysis of *EYA4* exon 13 shows the c.1177C>T change in an affected family member (III-2) and a normal control. The arrow indicates the changed base. (C) Multiple alignments of the amino acid sequence encoded by the *EYA4* gene including the HR domain in vertebrate species. The arrow marks the position of the p.Q393X mutation.

The genetic analysis of the *EYA4* gene identified a total of 4 sequence variants, including 2 nonsense mutations. A novel mutation, namely the single nucleotide substitution of a thymine to a cytosine at nucleotide position 1177 (c.1177C>T) in exon 13 was detected in the YS-151 family (Fig. 1A). This substitution resulted in a change in the codon at amino acid position 393 from a glutamine to a stop codon (p.Q393X), producing a truncated protein lacking the Eya VR domain (S1 Fig.). The affected family member (III-2) was heterozygous for this mutation (Fig. 1B). The PTA showed that the patient (III-2) suffered from moderate, bilateral hearing loss in low- and mid-frequencies and had increasing thresholds to profound hearing loss in the higher frequencies (Fig. 1A). A comparison of the amino acid sequence at the Eya HR domain showed that the partial region of EYA4 was highly conserved across multiple vertebrate species (Fig. 1C). This mutation was absent in all 100 normal controls, and none of the healthy controls in the 1000 Genomes Project carried this mutation. This evidence supports the hypothesis that it is a pathogenic mutation and causes hearing loss, rather than a rare polymorphism.

**Fig 2:**
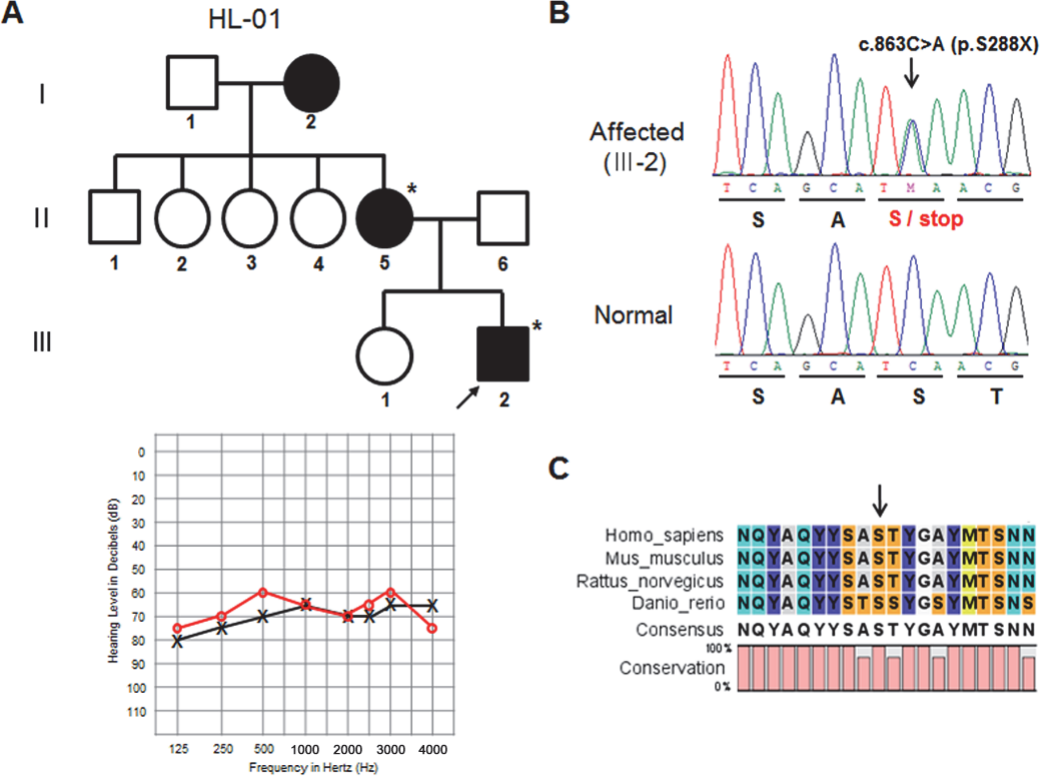
Mutation analysis of the *EYA4* gene in the HL-01 family. (A) Pedigree of the Korean family with autosomal dominant inheritance (upper panel). A three-generation pedigree including 10 members is presented. The filled symbols and open symbols indicate affected and unaffected individuals, respectively. The arrow designates the proband. Pure tone audiogram for the left and right ears of the HL-01 patient (III-2) (lower panel). The circles and crosses indicate unmasked air conduction thresholds for the right and left ears, respectively. (B) DNA sequencing analysis of *EYA4* exon 11 shows the c.863C>A change in an affected member (III-2) of the HL-01 family and a normal control. The arrow indicates the changed base. (C) Multiple alignments of the amino acid sequence encoded by the *EYA4* gene including the VR domain in vertebrate species. The arrow marks the position of the p.S288X mutation.

**Fig 3:**
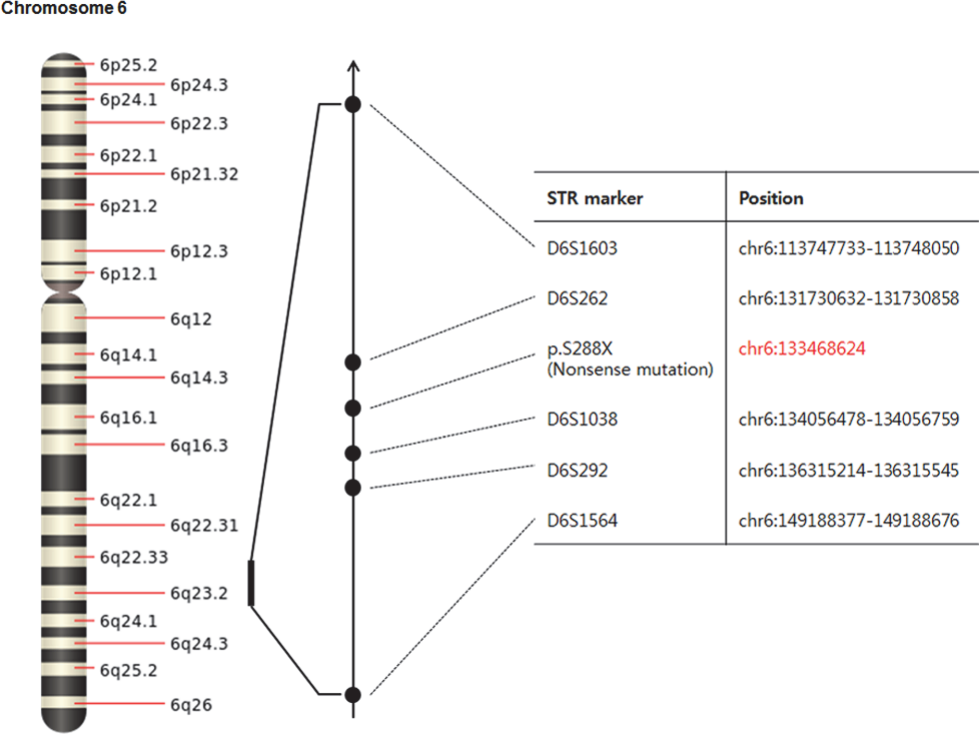
Location of the p.S288X mutation in *EYA4* and linked STR markers on chromosome 6q. The physical map marks the location of 5 microsatellite markers and positions.

The other nonsense mutation, identified in the HL-01 family (Fig. 2A), was a single nucleotide substitution of a cytosine for an adenine at nucleotide position 863 (c.863C>A) in exon 11. This substitution encodes a stop codon instead of a serine at amino acid position 288 (p.S288X), producing a truncated protein lacking the entire Eya VR domain. The affected family members (II-5 and III-2) were heterozygous for this mutation (Fig. 2A and B). The PTA of affected individual III-2 showed a flat audiogram in all frequencies in the moderate to severe range (Fig. 2A). A comparison of the amino acid sequence of the Eya VR domain partial region showed that p.S288X is highly conserved among various vertebrate species (Fig. 2C). The p.S288X mutation was previously identified in a Korean family labeled KNUF24 [13]. Therefore, we analyzed p.S288X-linked microsatellite markers to investigate whether the p.S288X allele in Koreans was a founder mutation. Five additional markers (D6S1603, D6S262, D6S1038, D6S292, and D6S1564) spanning 35.4 cM across the chromosome 6q23 region were selected and analyzed (Fig. 3). The affected individual II-5 of the KNUF24 family carried p.S288X (Table 2). The unaffected individuals (II-2 and III-2) of the KNUF24 family are her sister and daughter, respectively. Even though we could not construct haplotypes due to a lack of parental DNA samples to determine linkage, the markers better delineated the candidate region. The EYA4 gene was located between the D6S262 and D6S1038 microsatellite markers. While the affected subject (II-5 of KNUF24) was homozygous for the 178 allele at D6S262, the other affected subjects (II-5 and III-2 of HL-01) were homozygous for the 176 allele in the same region. All patients were found to be at least heterozygous for the 147 allele for D6S292, but the rest of the microsatellite markers were not clearly differentiated between affected and unaffected subjects.

**Table 2:** Microsatellite analysis of subjects carrying p.S288X and their family members.

	KNUF24*			HL-01^#^	
	II-2	II-5	III-2	II-5	III-2
D6S1603	263 263	261 263	261 265	263 265	261 265
D6S262	176 176	178 178	178 178	176 176	176 176
**p.S288X**	C C	C **A**	C C	C **A**	C **A**
D6S1038	181 181	181 181	181 181	177 181	181 181
D6S292	151 153	147 149	143 149	145 147	145 147
D6S1564	240 240	240 242	240 242	240 240	226 240

* KNUF24 is the family of previously study of Baek *et al*. (2012).

II-2, unaffected individual; II-5, affected individual; III-2, unaffected individual.

^#^ HL-01 is the family presented in this study.

II-5, affected individual; III-2, affected individual.

In addition, two single nucleotide substitutions were identified in this study (Table 3). The non-synonymous variant c.829G>A was discovered in 42 heterozygous patients and 7 homozygous patients. The allele frequency of adenine in the NCBI database (dbSNP) was 39% in the European and Asian populations. The other was a novel synonymous variant (c.417C>T) and was found in one heterozygous patient. Although this synonymous variant does not influence protein coding directly, it is possible that it regulates pre-transcription processes such as splicing patterns and splicing factor binding. We used two programs to predict the effect of splicing enhancers or silencers. The *EYA4* sequence variant was predicted to inhibit binding of a splicing enhancer by one of the two prediction programs (S1 Table). The PhyloP scores that determines the grade of conservation showed −0.27, which means that this site predicted to be fast-evolving. In the c.417C>T substitution, the SF2/ASF motif, one of four SR proteins used to identify ESEs, indicated a lower threshold value compared to the wild type, and the SC35 motif had a lower threshold value.

**Table 3:** SNPs of the *EYA4* gene identified in this study.

Location	Nucleotide change	Amino acid change	Heterozygous (n = 87)	Homozygous (n = 87)	Minor allele frequency	SNP ID
Exon 7	c.417C>T	p.S139S	1	0	-	Novel
Exon 11	c.829G>A	p.G277S	42	7	0.3933 (A)	rs9493627

### Genetic analysis of *GRHL2*

In the *GRHL2* gene, a total of 4 substitutions (c.1152G>A, c.1243G>A, c.1572A>G, and c.1722C>T) were identified (Table 4). The variant c.1243G>A was missense mutation that resulted in a change in the codon at amino acid position 415 from a valine to a isoleucine. This variant with a minor allele frequency of 2.98% in public and internal databases was predicted to be benign or likely benign by ClinVar and to be tolerated (score was 0.27) by SIFT, while this variant was predicted to be possibly damaging (score was 0.880) by PolyPhen-2 and to be disease-causing by MutationTaster. The variant c.1722C>T was absent from both the dbSNP of NCBI (http://www.ncbi.nlm.nih.gov/) and the 1000 genomes (http://www.1000genomes.org) databases. This variant was predicted to form new splicing enhancer sites by both programs (S1 Table). The PhyloP scores showed −3.623, which means that this site predicted to be fast-evolving like EYA4 c.417C>T. Using ESEfinder, the two motifs SRp40 and SRp55 of the SR proteins were predicted to bind the new site. The RESCUE-ESE program also predicted a new binding site. The other three variants have been reported with minor allele frequencies of 42.9%, 2.98%, and 3.76% (rs2287854 for c.1152G>A, rs3779617 for c.1243G>A, and rs34213258 for c.1572A>G, respectively).

**Table 4:** SNPs of the *GRHL2* gene identified in this study.

Location	Nucleotide change	Amino acid change	Heterozygous (n = 87)	Homozygous (n = 87)	Minor allele frequency	SNP ID
Exon 9	c.1152G>A	p.V384V	1	0	0.429 (G)	rs2287854
Exon 9	c.1243G>A	p.V415I	14	3	0.0298 (A)	rs3779617
Exon 13	c.1572A>G	p.P524P	9	0	0.0376 (G)	rs34213258
Exon 15	c.1722C>T	p.P574P	1	0	-	Novel

## Discussion

The EYA proteins are transcriptional coactivators that interact with the transcription factors SIX and DACH, but lack a DNA-binding domain [23]. The Eya HR domain of EYA4 is essential for the interaction with SIX3 [24]. To date, eight mutations have been reported in the *EYA4* gene, and other than one missense mutation, six result in truncated proteins that partially or completely lack the SIX3-interacting region in the Eya HR domain. In this study, the *EYA4* gene was screened in Korean patients with autosomal dominant NSHL, and two nonsense mutations, p.S288X and p.Q393X, were found. The p.Q393X mutation was first identified in this study, while p.S288X has previously been found in a Korean family [13]. The novel nonsense mutation p.Q393X leads to a truncated protein lacking the entire Eya HR domain, similar to other previously reported mutations. This suggests that this nonsense mutation may cause a similarly interrupted interaction with SIX3 due to the deletion of the Eya HR domain. As a result, this mutation may cause NSHL via its interaction with SIX3.

On the other hand, the EYA4 protein was partially functional. In a functional study of heterozygotes *in vitro*, the mutation causing the shortest truncation, c.1026_1027dupAA of EYA4, caused NSHL, but maintained the interaction between *EYA4* and the SIX1 and SIX2 proteins [4]. However, the shorter E193 (causing the deletion of a 4846-bp region that includes the last nucleotide of exon 9, intron 9, exon 10, and part of intron 10) caused NSHL and cardiomyopathy because these mutated proteins did not bind SIX proteins and could not dimerize with full-length EYA4 [9]. This shows that interruption of the EYA4 interaction with SIX1 and SIX2 impairs cardiac function, resulting in cardiomyopathy. The designed version E193 deletes not only the Eya HR domain but also the Eya VR domain. As a result, E193 causes dilated cardiomyopathy, unlike the other mutations [9]. In this study, the two nonsense mutations p.Q393X and p.S288X were expected to cause hearing loss but not cardiomyopathy, because they retain the Eya VR domain.

The p.S288X mutation was previously identified in a Korean family [13]. Therefore, we analyzed p.S288X-linked microsatellites to investigate whether the p.S288X allele in Koreans was a founder mutation. Although affected persons did not share the D6S1603 and D6S262 microsatellite regions, they shared the D6S1038 and D6S292 microsatellite regions. This suggests that the allele would be shortened. If the founder mutation was initiated a long time ago, the proportion of the haplotype was shortened due to genetic recombination. However, we could not rule out the possibility that the p.S288X mutation is in a mutational hotspot.

We compared clinical information for the patients in all *EYA4* mutation studies, including this study, and confirmed a link between clinical parameters and two domains of *EYA4*. The PTAs of patients with *EYA4* mutations were diverse, indicating that PTA was not associated with any of the truncated domains. This means that all mutations described to date in *EYA4*-related autosomal dominant NSHL cases were not consistent with a genotype-phenotype correlation between the mutated domains and the respective hearing loss patterns. We have performed a sensitivity analysis by taking the population frequency of mutations and identified in Koreans to be 5.4% (the upper limit of the 95% exact confidence interval of the mutation frequency estimated from the controls).

The GRHL family members are involved in otic development and hearing in animals, but an association with hearing loss in humans was found only in *GRHL2*, the causative gene of the DFNA28 locus [16]. In this study, 4 variations were detected in the *GRHL2* gene. The missense variant c.1243G>A was predicted to be possibly damaging by PolyPhen-2 and to be disease-causing by MutationTaster. However, this variant may not be a pathogenic mutation, because the 14 patients were heterozygous and 3 patients were homozygous for this variation of *GRHL2* gene caused autosomal dominant NSHL in this study and the adenine allele frequency in the NCBI (dbSNP) was high as 2.98%. The novel synonymous variant c.1722C>T was identified in 1 patient in this study. Because ESE prediction programs estimate that this variation may make a new ESE at this variant site, we were unable to exclude the possibility that it is a pathogenic mutation. In this case, functional studies must be carried out, and other variants were not predicted to affect hearing loss.

In conclusion, we confirmed that two transcription factors in 87 Korean patients caused autosomal dominant NSHL. We found 2 nonsense pathogenic mutations and 2 variants in the *EYA4* gene, and 1 novel variant and 3 polymorphisms in the *GRHL2* gene. These results provide fundamental information to predict the primary cause of NSHL in Korean patients and will be useful for *EYA4* or *GRHL2*-related hearing loss diagnosis in Koreans.

## Supporting Information

S1 Fig The schematic diagram of the coding region in the EYA family genes. A diagram of the EYA protein shows the two domains and features with early truncated mutant EYA proteins. The arrow designates the mutations that were identified in a previous study. The schematic shows p.S288X and p.Q393X mutations, which make truncated proteins at each position.
(TIF)

S1 Table *In silico* predictions of splicing regulatory element modifications for the novel *EYA4* variation. (DOCX)
