# Supplementary figures and images for: Evaluation of the Contribution of the EYA4 and GRHL2 Genes in Korean Patients with Autosomal Dominant Non-Syndromic Hearing Loss
Source: PLoS One. 2015 Mar 17;10(3):e0119443. doi: 10.1371/journal.pone.0119443 (PMC4363478; doi:10.1371/journal.pone.0119443)

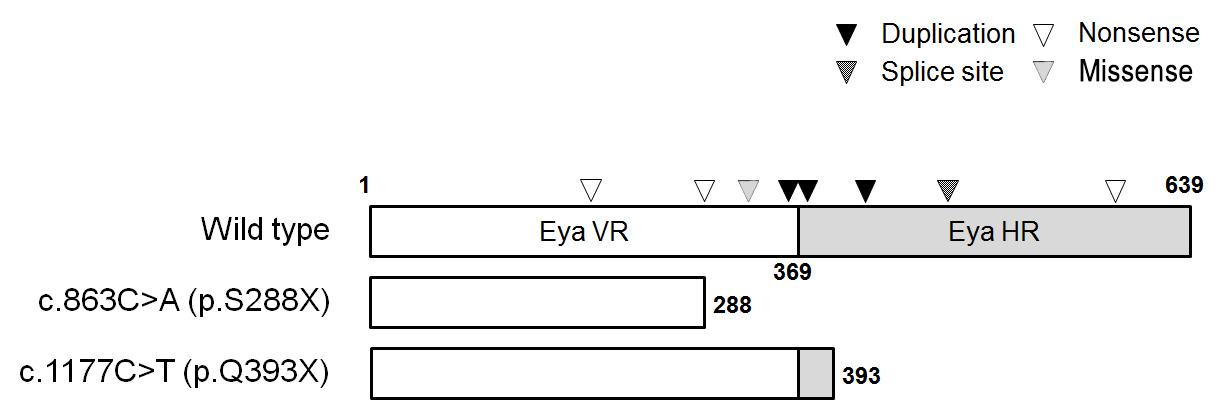

Supplement: S1 Fig — A diagram of the EYA protein shows the two domains and features with early truncated mutant EYA proteins. The arrow designates the mutations that were identified in a previous study. The schematic shows p.S288X and p.Q393X mutations, which make truncated proteins at each position. (TIF) [file pone.0119443.s001.tif]
