# Supplementary material for: Evaluation of the Contribution of the EYA4 and GRHL2 Genes in Korean Patients with Autosomal Dominant Non-Syndromic Hearing Loss
Source: PLoS One. 2015 Mar 17;10(3):e0119443. doi: 10.1371/journal.pone.0119443 (PMC4363478; doi:10.1371/journal.pone.0119443)
Supplement: S1 Table — (DOCX) [file pone.0119443.s002.docx]

**Supplementary table 1.** *In silico* predictions of splicing regulatory element modifications for the novel *EYA4* variation

|  |  | ESEfinder | | | RESCUE-ESE | |
| --- | --- | --- | --- | --- | --- | --- |
|  | PhyloP^*^ | Motif | Sequence (wt^**^/variant) | Score (wt/variant) | Sequence | Match (wt/variant)^****^ |
| *EYA4* exon 7  c.417C>T | -0.27 | SF2/ASF  (IgM-BRCA1) | **(C/T)**CCACAG | 2.50585/b.t.^***^ | No change | |
|  |  | SC35 | ATT**(C/T)**CCCA | 2.38864/b.t. |  |  |
| *GRHL2* exon 15  c.1722C>T | -3.623 | SRp40 | CC**(C/T)**GTGG | b.t./2.95038 | TGTGGA | 0/1 |
|  |  | SRp55 | **(C/T)**GTGGA | b.t./3.40507 |  |  |

^*^PhyloP, values between -14 and +6, to be conserved (positive scores) or to be fast-evolving (negative scores).

^**^wt, wild type; ^***^b.t., below threshold; ^****^Match, presence (1) or absence (0) of putative ESEs.

We used the default threshold values determined by the developers of the ESEfinder algorithm: 1.956 for SF2/ASF, 1.867 for SF2/ASF(IgM-BRCA1), 2.383 for SC35, 2.67 for SRp40, and 2.676 for SRp55.
